# Physically Active Lifestyle Attenuates Impairments on Lung Function and Mechanics in Hypertensive Older Adults

**DOI:** 10.3390/arm92040027

**Published:** 2024-07-22

**Authors:** Maysa Alves Rodrigues Brandao-Rangel, Boris Brill, Edilson de Souza Carvalho, Dobroslav Melamed, Renilson Moraes-Ferreira, Anamei Silva-Reis, Patricia Sardinha Leonardo, Claudio Ricardo Frison, Kátia De Angelis, Rodolfo P. Vieira

**Affiliations:** 1 Federal University of Sao Paulo (UNIFESP), Post-graduation Program in Sciences of Human Movement and Rehabilitation, Avenida Ana Costa 95, Santos 11060-001, SP, Brazil; maysarangel_4@hotmail.com (M.A.R.B.-R.); renilsonmoraesferreira@gmail.com (R.M.-F.); claudiofrison@gmail.com (C.R.F.); 2 Leniado Medical Center, Divrei Khayim St 16, Nethanya 4244916, Israel; drborisbrill@gmail.com; 3 Evangelical University of Goias (Unievangelica), Post-graduation Program in Humam Movement and Rehabilitation and in Pharmaceutical Sciences, Pharmacology and Therapeutics, Avenida Universitária Km 3,5, Anápolis 75083-515, GO, Brazil; edilsonsouza.carvalho@hotmail.com (E.d.S.C.); anameisreis97@gmail.com (A.S.-R.); pssardinha@yahoo.com.br (P.S.L.); 4 LibiPharm, Department of Research and Development, Derech Ben Gurion 70, Rehovot 7639461, Israel; dobroslav.melamed@gmail.com; 5 Federal University of Sao Paulo (UNIFESP), Department of Physiology, Rua Botucatu 862, São Paulo 04023-901, SP, Brazil; prof.kangelis@uninove.br

**Keywords:** hypertension, physical exercise, respiratory system, healthy lungs

## Abstract

**Highlights:**

This is the first study demonstrating that systemic arterial hypertension in older adults negatively impacts not only lung function and mechanics. In addition, this study shows that a physically active lifestyle prevents the decline of lung function and mechanics in older adults.

**What are the main findings?**

- Systemic arterial hypertension negatively affects not only the lung function but also lung mechanics.
- A physically active lifestyle by older adults prevents the impairment of lung function and mechanics induced by systemic arterial hypertension.

**What is the implication of the main finding?**

- Medical doctors who diagnose their patients, especially older adults with systemic arterial hypertension, should also check for lung function and mechanics.
- Medical doctors should advise their patients with systemic arterial hypertension to keep an active lifestyle to avoid hypertension-induced loss of lung function.

**Abstract:**

Aim: Physical activity attenuates hypertension in older adults, but its impact on pulmonary function and mechanics in hypertensive older adults is unknown. The study seeks to understand whether a physically active lifestyle can improve respiratory capacity, the mechanical efficiency of the lungs, and, consequently, the quality of life of these individuals, comparing data between groups of active and sedentary hypertensive older adults. Methods: This is a cross-sectional study. We evaluated 731 older adults, stratified into two initial groups: hypertensive older adults (HE; n = 445) and non-hypertensive older adults (NHE; n = 286). For a secondary analysis, we used the International Physical Activity Questionnaire to sub-stratify HE and NHE into four groups: physically inactive hypertensive (PIH; n = 182), active hypertensive (AH; n = 110), physically inactive non-hypertensive (PINH; n = 104), and active non-hypertensive (ANH; n = 65). Lung function was measured by spirometry, and lung mechanics were assessed by impulse oscillometry. Results: Hypertensive older adults presented reduced lung function compared to non-hypertensive older adults, and physical inactivity accentuated this decline. Regarding pulmonary mechanics, hypertensive older adults had higher resistance of the entire respiratory system (R5 Hz), the central airways (R20 Hz), and peripheral airways (R5–20 Hz), which may trigger bronchoconstriction. Conclusions: Hypertension is associated with impaired lung function and mechanics in older adults, and a physically active lifestyle attenuates these dysfunctions.

## 1. Introduction

Systemic arterial hypertension (SAH) is a prevalent chronic condition in humans, impacting over 1 billion individuals globally [1]. The complications, such as stroke, coronary artery disease, heart failure, and kidney disease, are leading reasons for illness and death, having a major impact on public health [1]. As the global population ages, older adults (≥65 years old) who are affected by hypertension are at a higher risk of suffering from organ damage, such as cardiovascular disease. Lowering blood pressure can help prevent and decrease complications related to SAH [1]. Yet managing hypertension in older adults poses challenges as many trials have excluded older individuals or have not reported results specific to this age group [2,3]. Furthermore, there is a wide variation in the global occurrence of hypertension among individuals aged 60 and above, with estimates ranging from 60% to 80% in numerous nations [3]. This notable frequency demonstrates how blood pressure tends to rise as people get older, along with the risks that come with aging [3]. Taking into account regional, genetic, lifestyle, and access to health care factors, it is crucial to acknowledge that prevalence can vary [2,3]. Prior research has shown that lung function is closely connected to its capacity to expand and revert to its original state, and the aging process is closely linked to incremental alterations in the mechanics of the respiratory system, leading to a decline in lung function [4]. Although tissue mechanical properties are crucial for lung and cellular function, there is limited understanding of microscale changes in lung mechanics across anatomical compartments throughout normal human aging. In this view, possible mechanisms include the following: A sedentary lifestyle can negatively affect lung health, decreasing lung capacity in hypertensive older adults through several interrelated mechanisms [5]. For instance, a sedentary lifestyle leads to decreased physical activity, which is essential for maintaining the strength and elasticity of the respiratory muscles [5]. In hypertensive older adults, lack of exercise can result in a reduction in lung capacity and respiratory efficiency [6]. In addition, such a sedentary lifestyle increases the risk of lung diseases, such as chronic obstructive pulmonary disease (COPD) and pneumonia [7]. Furthermore, hypertension can aggravate these conditions due to increased heart work and pressure on the pulmonary blood vessels [2]. Lastly, increased systemic inflammation is a hallmark linking hypertension and lung dysfunction, as a sedentary lifestyle results in higher levels of systemic inflammation, which, through pro-inflammatory, pro-fibrotic, free radicals, and other mediators, negatively affects the lungs, and exacerbates conditions such as asthma and COPD [8]. In the past few years, there has been a worldwide rise in acute respiratory tract infections and their associated complications, with a corresponding increase in the annual rate of pneumonia among individuals aged 60 and above in multiple countries^9^. Additionally, acute respiratory conditions are the primary reason for hospitalization in individuals with chronic health issues [9]. Engaging in consistent exercise has been proven to provide various health advantages, especially in reducing the harmful impacts of long-term illnesses [6,7]. More precisely, engaging in physical activity can boost cardiovascular health by decreasing blood pressure, enhancing endothelial function, and reducing inflammation [10,11]. It also has a crucial function in supporting lung health by increasing lung capacity, strengthening respiratory muscles, and facilitating effective gas exchange [11,12]. Nevertheless, the precise effects of physical exercise on lung function and mechanics in older adults with hypertension have not been fully studied. This research aims to address this gap by investigating if staying active can lessen the negative effects on lung function and mechanics that come with aging and hypertension.

There are various ways in which being physically active can improve lung function and mechanics. Physical activity has been proven to promote the production of anti-inflammatory cytokines and enhance markers of oxidative stress, in turn safeguarding lung tissues from harm [11,12]. In addition, consistent exercise improves respiratory muscle function, resulting in improved coordination between ventilation and blood flow in the lungs [11,12]. These physical changes can greatly help older adults with high blood pressure, as they are more prone to heart and lung issues. Even though the advantages of physical activity for heart and general health are widely known, there is still a substantial lack of research on how it impacts the respiratory system of elderly individuals with high blood pressure. Many previous studies have concentrated on younger individuals or those without chronic illnesses, resulting in a significant gap in our knowledge of how exercise programs can be customized to help elderly people with hypertension. The current research seeks to discover valuable insights through exploring this region, which can help in developing public health strategies and clinical practices to enhance the quality of life and health outcomes for this vulnerable group. With the increasing occurrence of hypertension and respiratory illnesses in older individuals, it is crucial to explore comprehensive strategies that tackle various health issues at the same time. This study suggests that being physically active could be used as a non-drug treatment to reduce the negative impact of hypertension on lung function and mechanics in elderly individuals. By comparing older adults with and without hypertension, this study aims to explore how regular physical activity can positively impact pulmonary health, leading to enhanced care strategies for aging individuals.

The current study seeks to investigate whether systemic arterial hypertension can affect the lung function and mechanics of elderly individuals and determine whether a physically lifestyle may affect such effects. In addition, this research aims to fill the current knowledge gap and provide evidence-based suggestions for improving respiratory health in elderly individuals with hypertension through physical activity.

## 2. Materials and Methods

All subjects read, agreed, and provided written informed consent prior to participating in the study. All experimental procedures used in the present study were approved by the institutional ethical committee at Nove de Julho University under number 1.021.635, in accordance with national recommendations for clinical studies and in compliance with the Declaration of Helsinki.

### 2.1. Study Design

This observational cross-sectional study enrolled older adult women and men from older adult houses (Older Adults Living Center), where they have access to various supervised sports activities, manual craftwork, medical consultations with geriatricians, and more, in the municipality of São José dos Campos, SP, Brazil. The inclusion criteria were as follows: (i) age ≥ 60 years (World Health Organization, 2015), (ii) absence of respiratory disease, (iii) diagnosis of hypertension by a specialized physician for hypertensive patients, (iv) non-smoking older adults, (v) absence of chronic degenerative, autoimmune, or neurological diseases, and (vi) controlled hypertension. Exclusion criteria included the following: (i) inability to correctly perform spirometry and oscillometry tests, and (ii) presence of infectious disease in the last 30 days.

Initially, 906 older adults were enrolled, and subsequently, 731 were classified into two groups: hypertensive older adults (HE; n = 445) and non-hypertensive older adults (NHE; n = 286). Unfortunately, of these 731 elderly people, only 461 were retained in the study, as 270 elderly people did not respond properly to the International Questionnaire of Physical Activity (IPAQ). Subsequently, these 461 elderly people were sub-classified based on their level of physical activity and distributed into four groups: physically inactive hypertensive (PIH, n = 182), active hypertensive (AH, n = 110), physically inactive non-hypertensive (PINH, n = 104), and active non-hypertensive (ANH, n = 65).The mean age of the elderly people was age = 69.88 ± 2.8.

Hypertensive older adults used the same medication with varying dosages, as it is part of the Brazilian national public health system (Sistema Único de Saúde—SUS; https://www.gov.br/saude/pt-br; accessed on 15 June 2024), which is entirely free in Brazil. The medications used included Atenolol, Hydrochlorothiazide, and Losartan Potassium, with doses individualized according to medical criteria.

### 2.2. Anthropometric Evaluations

Anthropometric measurements were performed using a flexible metal measuring tape with a resolution of 1 mm and a body weight scale with a stadiometer, providing a resolution of 0.1 kg and 1 mm for evaluating perimetry and body weight and height, respectively. Additionally, the body mass index (BMI) was calculated using the formula BMI = kg/m^2^ [11,12].

### 2.3. Blood Pressure Evaluation

Blood pressure measurements were conducted under specific conditions, including no recent exercise within the last 60 min, a resting period of 10 min, and abstinence from alcohol, smoking, or caffeine within the last 60 min. Patients were required to be free from strong emotions, pain, stress, or the use of adrenergic stimulant medications. The measurements were obtained with the patient seated, back supported, feet on the floor and uncrossed, and the arm at heart height, using an Omron^®^ digital automatic device model HEM 7122 (Omron Healthcare^®^, Osaki, Shinagawa-ku, Tokyo 141-0032, Japan) a digital arm blood pressure monitor validated by the Brazilian Society of Hypertension. At least two measurements were performed, with an interval of approximately two minutes, and the average of the last two measures was considered for analysis [11,12].

### 2.4. Lung Function and Lung Mechanics Evaluation

Lung function was assessed through spirometry using the Master Screen PFT Oscillometry system (Vyaire Medical^®^, Jaeger^®^, Höchberg, Germany). The parameters evaluated included FVC (forced vital capacity), FEV1 (forced expiratory volume in the first second), and FEV1/FVC (Tiffeneau index) using forced maneuvering, following the recommendations of the American Thoracic Society [13], with reference values for the Brazilian population [14]. Lung mechanics were evaluated through impulse oscillometry using the Masterscreen PFT Oscillometry system (Vyaire Medical^®^, Jaeger^®^, Höchberg, Germany) [12,15,16]. The measured parameters included R5Hz (total resistance of the respiratory system), R20Hz (resistance of proximal airways), X5Hz (reactance-elasticity of the lung tissue), and Z5Hz (respiratory system impedance). Results were presented both as a percentage of predicted values and in absolute units [12,15,16]. We clarify that both lung function and mechanics were performed before and after the administration of bronchodilator (400 mcg salbutamol sulfate).

### 2.5. Peripheral and Respiratory Muscle Strength Measurements

General muscle strength was evaluated using a hand grip dynamometer (Jamar^®^, Sammons Preston Rolyan, Boilingbrook, IL, USA) to assess hand grip strength [12,17], with results presented in kilograms (Kg). Respiratory muscle strength was evaluated using a manovacuometer (MVD-300 V.1.1 Microhard System^®^, Globalmed^®^, Porto Alegre, Brazil) to measure maximal inspiratory (MIP) and expiratory (MEP) pressure [12,17], with results presented in cmH_2_O.

### 2.6. International Physical Activity Questionnaire (IPAQ)

The International Physical Activity Questionnaire (IPAQ) is a standardized survey tool developed to measure physical activity levels across different populations and countries. The primary purpose of IPAQ is to provide a common instrument that can be used internationally to compare physical activity levels and to track changes over time. In addition, the IPAQ was used to assess the level of physical activity, categorized into four domains: work, transportation, domestic work, and leisure. Moderate activities were those performed for at least 10 min, causing an increase in breathing and heart rate, and inducing sweating. Vigorous activities produced greater increases in breathing, heart rate, and sweating. Individuals who reported <150 min of combined moderate and vigorous physical activity per week were considered insufficiently active [18].

### 2.7. Health-Related Quality of Life (HRQoL)

The SF-36 (Short Form Health Survey) is a widely used questionnaire designed to measure HRQoL. It was developed as part of the Medical Outcomes Study (MOS) to provide a comprehensive evaluation of an individual’s overall health and well-being. The SF-36 consists of 36 questions, which cover eight domains of health. These domains are as follows: physical functioning (PF), role physical (RP), bodily pain (BP), general health (GH), vitality (VT), social functioning (SF), role emotional (RE), and mental health (MH) [19].

### 2.8. Dyspnea Evaluation

The Medical Research Council (MRC) Dyspnea Scale is a widely used tool for assessing the severity of dyspnea, or breathlessness, in individuals with respiratory conditions. Dyspnea is a common symptom experienced by people with conditions such as COPD, asthma, interstitial lung disease, heart failure, and others. The MRC Dyspnea Scale provides a simple and standardized way to quantify the impact of dyspnea on a person’s daily activities. Key aspects of the MRC Dyspnea Scale are as follows:

Scale Description: The MRC Dyspnea Scale consists of five grades, each describing the level of breathlessness experienced by an individual during different activities.

Grade 1: Breathlessness only occurs with strenuous exercise.

Grade 2: Breathlessness occurs when walking up a slight hill or hurrying on level ground.

Grade 3: Walks slower than people of the same age on level ground because of breathlessness or need to stop to catch breath when walking at own pace.

Grade 4: Stops for breath after walking about 100 yards or after a few minutes on level ground.

Grade 5: Too breathless to leave the house or breathless when dressing or undressing.

This scale was used to assess the severity of dyspnea in patients with respiratory or cardiopulmonary conditions. Prognostic Indicator: Higher MRC grades are associated with increased morbidity, mortality, and reduced quality of life. Treatment Monitoring: Helps clinicians monitor changes in dyspnea severity over time and assess the effectiveness of interventions [8].

### 2.9. Statistical Analysis

Statistical analysis was conducted using GraphPad Prism 5.0 software. For comparisons between the HE and NHE groups, the non-paired T-test was employed, while for comparisons among the four sub-groups, two-way ANOVA was used, considering the two interfering factors (hypertension and physical activity), followed by Dunns’s post hoc test. A *p*-value < 0.05 was considered statistically significant.

## 3. Results

From 906 older adults initially recruited, 731 who fulfilled the inclusion and exclusion criteria were classified into two groups: hypertensive older adults (HE; n = 445, age = 69.88 ± 2.8, weight (kg) 70.28 ± 4.2, height 1.60 ± 2.2, BMI (kg/m^2^) 28.6 ± 2.9, systolic blood pressure (mmHg) 150.7 ± 1.4, diastolic blood pressure (mmHg) 89.4 ± 0.7, waist circumference (cm) 90.13 ± 3.7) and non-hypertensive older adults (NHE; n = 286, age = 67.97 ± 0.7 weight (kg) 68.65 ± 2.1, height 1.58 ± 4.6, BMI kg/m^2^) 28.3 ± 2.4, systolic blood pressure (mmHg) 114.3 ± 0.7, diastolic blood pressure (mmHg) 74.7 ± 0.2, waist circumference (cm) 72.65 ± 1.7. In addition, no differences were found among the groups regarding the analysis of dyspnea, as evaluated by the Medical Research Council (MRC) dyspnea scale (*p* > 0.05).

For a second analysis on the effects of the levels of physical activity, volunteers were distributed into four groups: physically inactive hypertensive (PIH, n = 182), active hypertensive (AH, n = 110), physical inactive non-hypertensive (PINH, n = 104), and active non-hypertensive (ANH, n = 65). The characterization of the groups is presented in Table 1. In addition, there are no differences between the parameters demonstrated in Table 1 comparing the four groups, except for systolic blood pressure (SBP), where ANH presented lower SPB compared to PI (*p* < 0.05), AH (*p* < 0.05) and PINH (*p* < 0.05) group. Similarly, maximal inspiratory pressure (MIP) was higher in AH and ANH in comparison to PIH and PINH groups (*p* < 0.05).

**Figure 1:**
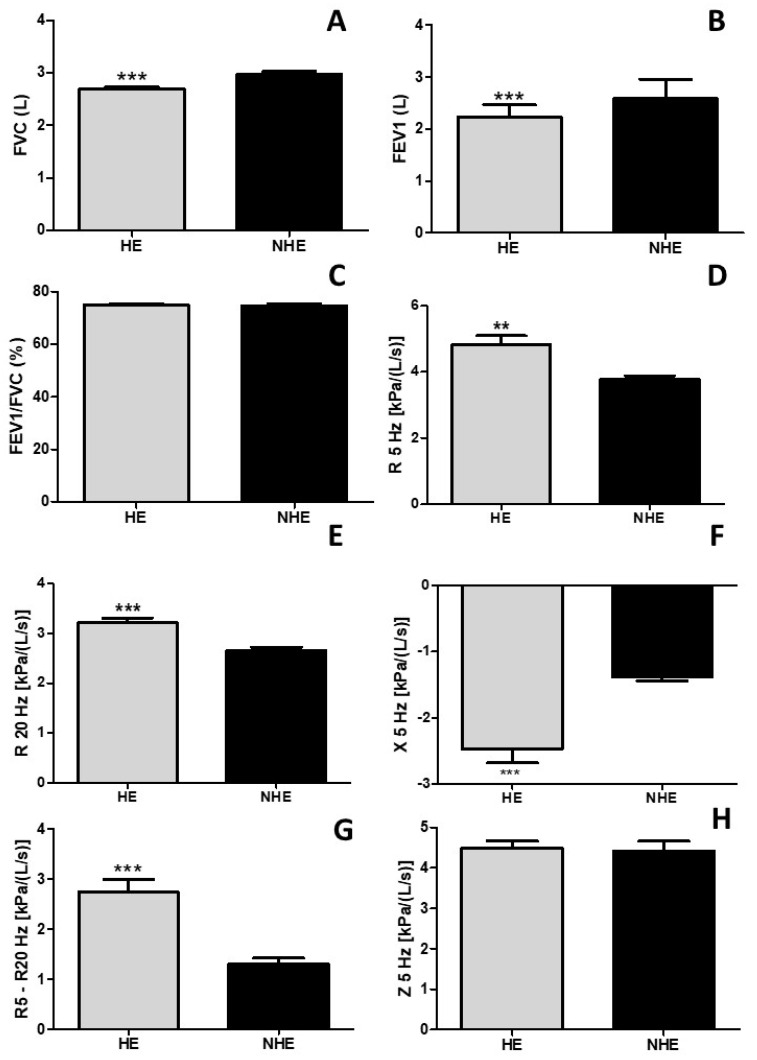
FVC (forced vital capacity), FEV 1 (forced expiratory volume in the first second) and FEV1/FVC (Tiffeneau index), R5Hz (total resistance of respiratory system), R20 Hz (resistance of proximal airways), X5Hz (reactance-elasticity of the lung tissue), and Z5Hz (respiratory system impedance). HE = hypertensive older adults; NHE = non-hypertensive older adults. ** *p* < 0.01; *** *p* < 0.001. Panel (**A**) shows the forced vital capacity (FVC). Panel (**B**) shows the forced expiratory volume in the first second (FEV1). Panel (**C**) shows the FEV1/FVC ration. Panel (**D**) shows the resistance of the whole respiratory system (R5 Hz). Panel (**E**) shows the resistance of proximal airways (R20 Hz). Panel (**F**) shows the reactance/elasticity of the lungs (X5 Hz). Panel (**G**) shows the resistance of distal airways (R5–R20 Hz). Panel (**H**) shows the impedance of the respiratory system (Z5 Hz). ** *p* < 0.01; *** *p* < 0.001.

Figure 1 shows that pulmonary function and mechanics of older adults with hypertension (HE) are impaired in relation to non-hypertensive (NHE) older adults. Figure 1A shows that the HE group presented reduced forced vital capacity (FVC %) in comparison to the NHE group (HE vs. NHE, *p* < 0.0001). Similarly, Figure 1B shows that the HE group presented reduced forced expiratory volume in the first second (FEV1 %) in comparison to the NHE group (HE vs. NHE, *p* < 0.0001). However, the FEV1/FVC values (% predicted) did not present differences between the groups. Regarding the pulmonary mechanics, the results showed in Figure 1D revealed that the HE group presented increased resistance of the whole respiratory system (R5Hz) (HE vs. NHE, *p* < 0.01), while Figure 1E shows similar results for the proximal airways (R20Hz) (HE vs. NHE, *p* < 0.001) and Figure 1G for distal airways (R5Hz–R20Hz) (HE vs. NHE, *p* < 0.001). Furthermore, Figure 1F shows that X5Hz (reactance-elasticity of the lung tissue) was increased in the HE group in comparison to the NHE group (HE vs. NHE, *p* < 0.001), while in Figure 1H, no differences were observed among the HE vs. NHE groups (*p* > 0.05) for Z5Hz (respiratory system impedance).

**Figure 2:**
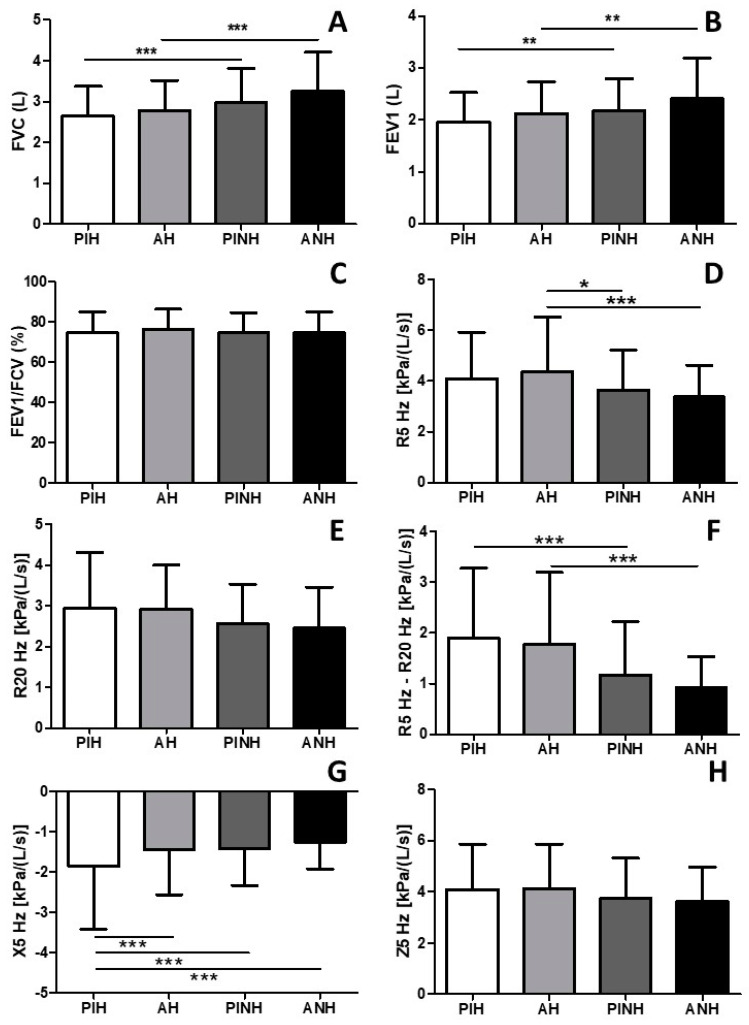
FVC (forced vital capacity), FEV 1 (forced expiratory volume in the first second) and FEV1/FVC (Tiffeneau index), R5 Hz (total resistance of respiratory system), R20 Hz (resistance of proximal airways), X5 Hz (reactance-elasticity of the lung tissue), and Z5 Hz (respiratory system impedance). HE = hypertensive older adults; NHE = non-hypertensive older adults. Panel (**A**) shows the forced vital capacity (FVC). Panel (**B**) shows the forced expiratory volume in the first second (FEV1). Panel (**C**) shows the FEV1/FVC ration. Panel (**D**) shows the resistance of the whole respiratory system (R5 Hz). Panel (**E**) shows the resistance of proximal airways (R20 Hz). Panel (**F**) shows the resistance of distal airways (R5–R20 Hz). Panel (**G**) shows the reactance/elasticity of the lungs (X5 Hz). Panel (**H**) shows the impedance of the respiratory system (Z5 Hz). * *p* < 0.05; ** *p* < 0.01; *** *p* < 0.001.

Figure 2 shows the effects of physically active lifestyle on pulmonary function and mechanics of the older adults with and without hypertension. In Figure 2A, we observed that the physically inactive hypertensive (PIH) group presented a reduced FVC when compared to the physically inactive non-hypertensive (PINH) group (*p* < 0.001), and the active hypertensive (AH) group presented a reduced FVC when compared to the active non-hypertensive (ANH) group (*p* < 0.001). Similarly, we observed in Figure 2B that PIH presented a reduced FEV1 when compared with PINH (*p* < 0.01), as well as AH vs. ANH (*p* < 0.01). These results reinforce the information that systemic arterial hypertension significantly affects the lung function. On the other hand, no differences were observed among the four groups for the FEV1/FVC (Figure 2C; *p* > 0.05). The evaluation of lung mechanics, as shown in Figure 2D, revealed that the resistance of the whole respiratory system (R5Hz) was higher in the AH group in comparison to the PINH (*p* < 0.05) and ANH (*p* < 0.001) groups, while no differences were observed for the resistance of proximal airways R20Hz (Figure 2E; *p* > 0.05). Concerning the resistance of small airways (R5Hz–R20Hz), Figure 2F shows that the PIH presented higher resistance of small airways than the PINH group (*p* < 0.001) as well as the AH group in comparison to the ANH group (*p* < 0.001). Of note, Figure 2G showed that reactance (X5Hz; elasticity of the lung tissue) was higher in the PIH in comparison to the AH (*p* < 0.001), PINH (*p* < 0.001) and ANH (*p* < 0.001) groups. Lastly, Figure 2H shows that no differences were observed for Z5Hz (impedance of respiratory system).

Table 2 shows the results from IPAQ, SF-36 and MRC from all four groups. The results demonstrated that the groups physically inactive hypertensive (PIH; *p* < 0.001) and physically inactive non-hypertensive (PINH; <0.001) presented a low score of physical activity when compared to both active hypertensive (AH) and active non-hypertensive (ANH) groups. In addition, the analysis of quality of life assessed using the SF-36 questionnaire showed that the AH group presented better physical functioning compared to the PIH and PINH groups (*p* < 0.001). The analysis of bodily pain using the SF-36 questionnaire showed that the AH group presented better physical functioning compared to the PIH, PINH, and ANH groups (*p* < 0.001). The analysis of general health using SF-36 revealed that a better score for the ANH and AH groups compared to the PIH and PINH groups (*p* < 0.001). Similarly, a better score for the ANH and AH groups compared to the PIH and PINH groups (*p* < 0.001) was found for vitality by using SF-36. Considering the social functioning, according to SF-36, a worse score was found for the PIH group compared to the AH, PINH, and ANH groups (*p* < 0.001). For mental health, using SF-36, AH presented a better score compared to PIH, PINH, and ANH (*p* < 0.001). The analysis of dyspnea using the MRC dyspnea scale did not reveal any differences among the four groups (*p* > 0.05).

## 4. Discussion

The present study showed for the first time that SAH, beyond lung function, also impairs lung mechanics in older adults and that a physically active lifestyle is associated with attenuation of such impairments, while no differences were found regarding the levels of dyspnea.

Dyspnea is an imbalance between the demand and the ability to breathe. Approximately 30% of those aged 60 years or older report difficulty to breath while walking at a horizontal level or on a slope [18]. The five main etiologies for chronic dyspnea in the older adults include anemia, cardiovascular disease, physical deconditioning, psychological disorders, and respiratory diseases [20]. The present study showed that SAH did not significantly impair the respiratory symptom of dyspnea, perhaps because although a statistically significant reduction of FEV1 was observed, such reduction could not be strong enough to induce a persistent cough. In addition, this can be explained by the fact that the body seeks compensation for a balance for the ventilation/perfusion ratio mainly during aging [5,17,18,20,21] in the presence of a change that compromises pulmonary elasticity [5,17,21,22,23], which, in our study, was observed functionally by the impairment in the lung reactance (X5Hz) in hypertensive older adults. This impairment in the lung reactance supports the concept that a compensatory hyperventilation of pulmonary regions with normal functioning prevents the increase in CO_2_ in the arterial blood, where diffusion/perfusion ratio tends to be compensated for non-dyspnea status.

The complete lung mechanics characteristics were evaluated by impulse oscillometry system, which proved to be a great advantage in assessing pulmonary changes in older adults because it is easy, required the minimum patient cooperation, is quick (around 40 s of data collection), presents reproducible measurements [12,15,16,17], and it was accomplished by all the older adults. In addition, we observed, in hypertensive older adults, increased resistance of the whole respiratory system (R5Hz), proximal airways (R20Hz), and distal airways (R5Hz–R20Hz) and increased reactance (X5Hz) compared with non-hypertensive older adults (NHE). Of note, correlations between increased pulmonary resistance are classically related to reduced FEV1, demonstrating that increased pulmonary resistance results in airway obstruction (reduced FEV1) [12,15,16,17]. In this way, similarly to the lung mechanics analysis, the present study also observed that HE presented a greater decline in pulmonary function represented by reduced forced vital capacity (FVC) and forced expiratory volume in the first second (FEV1). These findings regarding the decline in the lung function in HE population agree with the findings from general population of hypertensive individuals, as demonstrated by Margretardottir et al. (2009), reinforcing that SAH, independent of the age of the population studied, induces impairment in the lung function, which can also be detected by changes in the lung mechanics analysis by using impulse oscillometry system [8]. In addition, the reduction in FVC observed in PIH (physically inactive hypertensive) group may be justified, at least partially, due to physical inactivity and by reduced MIP and MEP, which classically compromise FVC by limiting chest expansion and diaphragm movement [5,11,17,18,21,22].

In recent studies, the correlation between SAH and pulmonary function alterations has been described [4,8,17,18,20,21], although the mechanisms remain unknown. It is known that SAH is related to increased systemic and pulmonary vascular resistance, as well as increased vessel stiffness [1,2]. Due to the highly vascular nature of the lung and the intimate anatomical coupling of vascular parenchymal elements, it is quite possible that, regardless of any alteration of the pulmonary parenchyma, the loss of elasticity of the pulmonary vascular tree would adversely affect FVC and FEV1 [4,8,17,18,20,21]. In any case, the present study was able to demonstrate that hypertensive older adults presented both increased resistance of the whole respiratory system (R5 Hz) and proximal (R20 Hz) and dystal (R5–R20 Hz) airways beyond reduced FEV1, indicating that SAH induces impairment in the airway resistance, leading to reduced lung function, notably FEV1. In the same way, reduced FVC was also detected and reduced in hypertensive older adults in the present study. This finding agrees with other studies in which the authors demonstrated that hypertensive adults present reduction in vital capacity [4,8,17,18,20,21]. This confirmation (hypertensive older adults with reduced vital capacity) presents a correlation with increased (impaired) lung reactance (X5 Hz), which is being demonstrated for the first time. Thus, the results obtained in the present study showing impaired X5Hz in HE reinforces the concept that SAH definitively induces pulmonary alterations, while a physically active lifestyle may inhibit such alterations.

SAH is the most prevalent chronic disease worldwide, with a long asymptomatic course, which requires changes in lifestyle, adding to physical activity [6,7,10,19,21]. Therefore, the management of hypertension in sedentary older adults is an important challenge in healthcare, as hypertension is a significant risk factor for cardiovascular disease, stroke, and other chronic conditions. The optimal approach for these patients involves a combination of exercise physiology and medical strategies [21,22]. A multidisciplinary approach is essential for successful management of hypertension in sedentary older adults. Collaboration between exercise physiologists, physicians, nurses, and other healthcare professionals allows for the development of integrated, personalized care plans that address not only high blood pressure, but also other cardiovascular risk factors and the patient’s overall health [21,22]. In addition, there is a high frequency of comorbidities and adverse effects in the geriatric population that do not perform physical activities, especially in Chronic Obstructive Pulmonary Disease patients, who normally present SAH beyond pulmonary affection [23,24]. A very strong association was also observed between the presence of pulmonary problems with hypertensive older adults and physical inactivity. Impaired pulmonary function and mechanics in older adults’ people with hypertension suggest that the lungs are also a “target organ” of hypertension [7,10,21]. Our results propose that the association of physical inactivity with arterial hypertension remains significant and that the older adults who have hypertension and perform physical activity have more preserved lung function and mechanics. According to some studies, the levels of moderate regular physical activity in the older adults are associated with the improvement of pulmonary function and, eventually, protecting themselves from the decline of aging [7,8]. However, the study suggests for the first time that, in addition to impaired pulmonary function in older adults with SAH, the central and peripheral airway resistance, as well as the decreased peripheral elastic lung tissue properties represented by the values of (X5Hz-reactance), clearly demonstrate that SAH induced a restrictive and an obstructive pulmonary response, while the regular practice of physical activity, as denoted by a physically active lifestyle, inhibited such response.

The present study has some limitations: The study design does not allow deeper conclusions because it was performed at a single point of time, although it remains as a pioneering study into the understanding the pulmonary effects of systemic arterial hypertension and the involvement of a physically activity lifestyle in these effects. However, further research to understand the cellular and molecular mechanisms are necessary.

## 5. Conclusions

We conclude that systemic arterial hypertension compromises lung function and mechanics in older adults and that a physically active lifestyle seems to partially accentuate these impairments. Thus, having a physically active lifestyle can mitigate these hypertension-induced pulmonary alterations in older adults. Therefore, the results of this study highlight an important message for cardiologists: hypertensive patients may exhibit lung dysfunction even in the absence of respiratory symptoms.

## Figures and Tables

**Table 1:** Clinical and anthropometric volunteers’ characteristics.

	PIH (n = 182)	AH (n = 110)	PINH (n = 104)	ANH (n = 65)	*p* Value	F Value
Age (years)	70.07 ± 7.49	69.39 ± 5.49	67.42 ± 6.34	66.83 ± 5.18	*p* > 0.05	5.073
SBP (mmHg)	140.28 ± 16.07 *	144.44 ± 18.46 *	140.94 ± 21.35 *	129.75 ± 16.74	*p* < 0.05	9.084
DBP (mmHg)	75.96 ± 9.14	72.56 ± 18.12	79.61 ± 9.78	79.25 ± 8.06	*p* > 0.05	2.471
Weight (kg)	70.86 ± 15.15	68.76 ± 10.75	66.57 ± 13.89	67.52 ± 12.88	*p* > 0.05	2.084
Height (m)	1.56 ± 0.08	1.57 ± 0.07	1.57 ± 0.08	1.59 ± 0.10	*p* > 0.05	2.056
BMI (kg/m^2^)	26.92 ± 8.83	27.88 ± 4.34	26.76 ± 4.52	26.60 ± 3.53	*p* > 0.05	1.980
HGS left (kg)	22.50 ± 10.08	24.78 ± 7.38	24.07 ± 8.69	25.72 ± 9.95	*p* > 0.05	2.073
HGS right (kg)	23.37 ± 11.02	25.35 ± 7.45	24.99 ± 8.95	27.55 ± 10.51	*p* > 0.05	4.744
MIP (cm H_2_O)	50.69 ± 23.48	59.95 ± 22.90 ^#^	62.71 ± 28.46	69.68 ± 35.24 ^#^	*p* < 0.05	8.026
MEP (cm H_2_O)	64.66 ± 34.50	81.36 ± 42.92 ^&^	76.77 ± 49.82	82.13 ± 35.44 ^&^	*p* < 0.05	6.589
HC (cm)	96.59 ± 12.38	92.62 ± 10.90	90.79 ± 11.63	90.92 ± 10.58	*p* > 0.05	2.214
WC (cm)	104.58 ± 10.92	101.12 ± 14.77	100.19 ± 9.63	101.73 ± 8.23	*p* > 0.05	3.470
WHR	0.93 ± 0.09	0.90 ± 0.08	0.90 ± 0.08	0.89 ± 0.07	*p* > 0.05	2.811
PA (years)	-	4.04 ± 3.98	-	4.69 ± 4.13	*p* > 0.05	2.416

SBP: systolic blood pressure, DSP: diastolic blood pressure, BMI: body mass index, HGS: hand grip strength, MIP: maximal inspiratory pressure, MEP: maximal expiratory pressure, HC: hip circumference, WC: waist circumference, WHR: waist-to-hip ratio, PA: physical activity. * *p* < 0.05 comparing PIH, AH and PINH with ANH group. ^#^ *p* < 0.05 comparing AH with PIH group and ANH with PINH group. ^&^ *p* < 0.05 comparing AH with PIH group and ANH with PINH group.

**Table 2:** Shows the results for the IPAQ, SF-36, and MRC questionnaires.

	PIH (n = 182)	AH (n = 110)	PINH (n = 104)	ANH (n = 65)	*p* Value	F Value
IPAQ Physical activity time (min/week)	20.27 ± 8.71	184.83 ± 20.62 *	24.32 ± 10.46	258.76 ± 28.87 *	<0.001	9.98
SF-36 Physical functioning	62.84 ± 20.45	74.28 ± 23.30 *	67.14 ± 23.66	69.59 ± 22.27	<0.001	10.54
SF-36 Bodily pain	49.75 ± 29.85	63.45 ± 43.21 *	50.66 ± 30.94	36.29 ± 28.28	<0.001	15.66
SF-36 Role-physical	86.12 ± 29.97	87.59 ± 32.39	91.16 ± 26.42	92.85 ± 24.41	>0.05	0.8514
SF-36 General health	40.35 ± 22.88	55.80 ± 24.22 *	46.77 ± 23.37	60.59 ± 25.00 *	<0.001	20.11
SF-36 Vitality	40.86 ± 31.47	69.91 ± 27.35 *	55.04 ± 33.60	55.66 ± 32.05 *	<0.001	14.85
SF-36 Social functioning	46.87 ± 32.49 *	77.01 ± 30.75	63.2 ± 35.73	70.69 ± 33.42	<0.001	11.99
SF-36 Role-emotional	91.08 ± 26.13	86.93 ± 31.29	94.27 ± 22.74	98.03 ± 14.07	>0.05	2.954
SF-36 Mental health	38.71 ± 30.11	69.84 ± 27.21 *	55.07 ± 31.92	56.97 ± 31.25	<0.001	20.99
MRC Dyspnea scale	9.37 ± 2.54	9.89 ± 2.02	9.69 ± 2.13	9.23 ± 1.93	>0.05	2.132

For IPAQ * *p* < 0.001 AH and ANH compared to PIH and PINH groups. For physical functioning * *p* < 0.001 AH compared to PIH and PINH group. For bodily pain * *p* < 0.001 AH compared to PIH, PINH and ANH group. For general health * *p* < 0.001 ANH and AH compared to PIH and PINH. For general health * *p* < 0.001 ANH and AH compared to PIH and PINH. For vitality * *p* < 0.001 ANH and AH compared to PIH and PINH. For social functioning * *p* < 0.001 PIH compared to AH, PINH and ANH. For mental health * *p* < 0.001 AH compared to PIH, PINH and ANH.

## Data Availability

All raw data will be freely available upon a reasonable request to the corresponding author of this manuscript.
